# The impact of insomnia on anxiety and depression: a longitudinal study of non-clinical young Chinese adult males

**DOI:** 10.1186/s12888-023-04873-y

**Published:** 2023-05-24

**Authors:** Xiaofei Mao, Fan Zhang, Cun Wei, Ziqiang Li, Chenwei Huang, Zuoer Sun, Jianguo Zhang, Wenxi Deng, Tianya Hou, Wei Dong

**Affiliations:** 1 Faculty of Psychology, Naval Medical University, #800 Xiangyin Road, Shanghai, 200433 China; 2 School of Basic Medical Science, Naval Medical University, Shanghai, 200433 China

**Keywords:** Insomnia, Anxiety, Depression, Longitudinal study, Non-clinical young males

## Abstract

Insomnia, anxiety, and depression commonly co-occured and were closely related. Most of the prior studies were cross-sectional, with a poor ability to infer causality. Longitudinal study was needed to classify the relationships. The present study conducted a longitudinal study of non-clinical young Chinese males to investigate whether insomnia predicted the likelihood of future anxiety and depression, and vice versa. Convenient sampling method was applied, and 288 participants was recruited from Shanghai in October 2017 with Athens Insomnia Scale (AIS), Generalized Anxiety Disorder-7 (GAD-7) and Patient Health Questionnaire-9 (PHQ-9). 120 of them were re-tested in June 2018. The drop-out rate was 58.33%. Correlation analyses and cross-lagged analysis showed that AIS global score was significantly positively related with scores of depression and anxiety at baseline and follow-up. Insomnia was a predictive factor of anxiety, but it can’t predict depression. In sum, insomnia may be an important cause of anxiety, while no predictive relationship was found between insomnia and depression.

**Supplementary Information:**

The online version contains supplementary material available at 10.1186/s12888-023-04873-y.

## Introduction

As a widespread disease, Insomnia is usually defined as poor sleep or dissatisfaction, accompanied by serious pain, as well as social, interpersonal, and occupational problems [1–4]. With the increasingly fierce social competition, the incidence of insomnia, anxiety and depression of adults was gradually increasing, and the relationships among them were complex [5].

Insomnia, anxiety, and depression commonly co-occured and were closely related [5–7]. A large number of studies were conducted to explore the association of insomnia with anxiety and depression. A review by Taylor et al. concluded that insomnia was a strong risk factor for depression and anxiety [8]. Moreover, people with insomnia reported higher levels of anxiety and depression than those without insomnia and were 17.35 and 9.82 times as likely to have clinically significant anxiety and depression, respectively [9]. Patients with clinical insomnia had a higher risk of developing anxiety and depression than patients without insomnia, which highlighted the importance of insomnia in the development of anxiety and depression [10, 11]. These studies indicated bad sleep quality may have negative influence on depression and anxiety.

However, evidence also suggested that insomnia was bidirectionally related to anxiety and depression [7, 9–13]. Common-cause hypothesis was proposed to explain the bidirectional relationship between insomnia and anxiety / depression [14, 15], which hypothesized that insomnia and anxiety/depression were influenced by a common factor, such as neural substrates. According to the common-cause hypothesis, disruption in both anxiety / depression and sleep quality may appear when the common factor alters.

Given the complex relationships between insomnia and anxiety/depression, most of the previous studies are cross-sectional, and the ability to identify causal relationships is limited. More longitudinal studies are needed to confirm the causal relationship between insomnia and psychiatric illnesses [10, 16, 17]. Moreover, much of the evidence for the relationship is based on clinical samples [18]. However, relying solely on clinical samples may exaggerate the association of insomnia with anxiety and depression [19]. Therefore, it is necessary to conduct longitudinal research based on non-clinical samples.

Therefore, in order to overcome the shortcomings in the previous studies mentioned above, we conduct a longitudinal study among non-clinical young Chinese males to investigate whether insomnia predicts the likelihood of future anxiety and depression, and vice versa.

## Methods

### Sample size calculation

G^*^Power software version 3.1.9.2 was used to estimate required sample size of this study. The present study used hierarchical regression analysis to analyze interactions between insomnia, anxiety, and depression. Therefore, F-test (Linear multiple regression: Fixed model, *R*^*2*^ deviation from zero) was chosen and effect size (*f*^*2*^) was set at 0.15 [20–22], alpha value was set at 0.05. Approximately 68 participants would provide 80% power and 88 participants would provide 90% power to detect a statistical significance.

### Subjects and procedures

There were 2 inclusion criteria in this study. They were listed as follow: (1) no dyslexia, (2) Chinese male, and (3) aged 18 years old or above. The exclusion criterion was the experiences of being diagnosed with mental disorder. Convenient sampling method was applied to recruit subjects in Shanghai.

The respondents were asked to sit in a room and filled out all the paper questionnaires. All participants signed written informed consent form in ethics approval and consent to participate form. The study was approved by the ethical committee of Naval Medical University in accordance with the ethical standards established in the 1964 Declaration of Helsinki and its later amendments. Participants received gifts after completing the survey. All the subjects were assured their responses were anonymous and confidential and they were free to withdraw at any time without penalty.

**Fig. 1:**
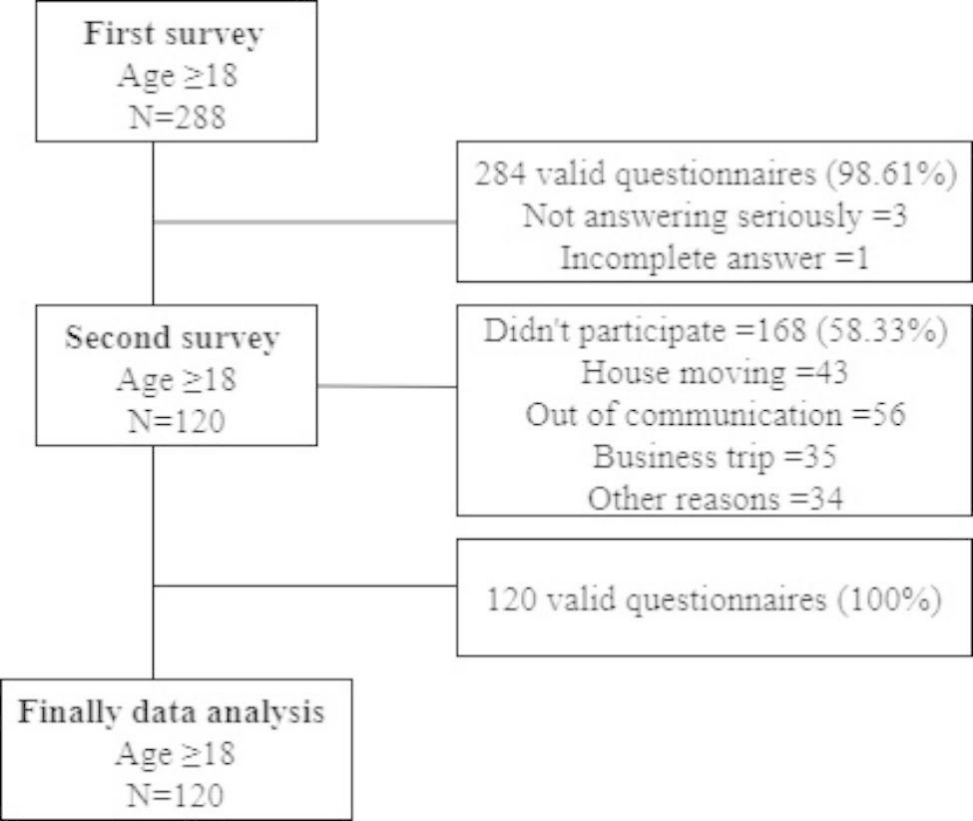
Flow Diagram of Sample Selection

A flow diagram of sample selection is presented in Fig. 1. 288 participants were recruited in non-clinical young Chinese male population in the first investigation (Time point 1, T_1_) with a response rate of 98.61% (284 valid questionnaires were received following the exclusion criteria). 120 of them participated in the second survey (Time point 2, T_2_) with a response rate of 100%. 168 participants didn’t complete the study due to house moving, out of communication, business trip and so on. Therefore, data of 120 subjects from 2 survey was analyzed. Participants were asked to respond to a questionnaire consisting of established instruments that assessed insomnia, anxiety, and depression in October 2017 (T_1_) and accepted a re-test 8 months later in June 2018 (T_2_). The average age of 120 subjects was 24.30 ± 4.47, and the average year of educational level was 14.03 ± 1.79.

### Measurement

#### Demographics

In the present study, demographic information including name, age, and educational level were recorded.

##### Athens insomnia scale, AIS

AIS was developed by Soldatos and colleagues to assess the severity of insomnia (including sleep induction, awakenings during the night, early morning awakening, total sleep time, overall quality of sleep, problems with sense of well-being, functioning, and sleepiness during the day) according to ICD-10 diagnostic criteria. It is a self-assessment psychometric tool in reliably establishing the diagnosis of insomnia which has previously shown high consistency, reliability, and external validity [23, 24]. AIS includes 8 items, and each item can be rated 0–3, with 0 corresponding to no problem at all and 3 to very serious problem. The responders are requested to rate each item positive only if they had experienced their sleep difficulty at least three times a week during the last month. Total score of AIS ranged from 0 to 24. A total score of 6 or more indicated that the participant illustrated insomnia symptoms. In the present study, the Cronbach’s alpha was 0.821 (T_1_) and 0.821 (T_2_).

##### Generalized anxiety disorder-7, GAD-7

The GAD-7 is a valid and efficient tool for screening GAD and assessing its severity in clinical practice and research [25]. The 7-item questionnaire was applied to ask participants how often they were bothered by each symptom during the last 2 weeks. Response options were “not at all”, “several days”, “more than half the days” and “nearly every day” scored as 0, 1, 2, and 3, respectively. The cut-off score for the diagnosis of anxiety is 10. In the present study, the Cronbach’s alpha was 0.871 (T_1_) and 0.819 (T_2_).

##### Patient health questionnaire-9, PHQ-9

The PHQ-9 includes 9 items pertaining to the DSM-IV criteria for depressive disorder [26]. Each item is rated on a 4-point scale from 0to 3 (0-never; 1- several days; 2-more than half the time; and 3-nearly every day) within the last two weeks before the completion of the survey. The cut-off score for the diagnosis of depression is 10. As long as item 9 appears (thoughts that you would be dead or of hurting yourself in some way), it is considered as positive, regardless of the number and duration of occurrence. In the present study, the Cronbach’s alpha was 0.784(T_1_) and 0.826(T_2_).

### Data analysis

**Fig. 2:**
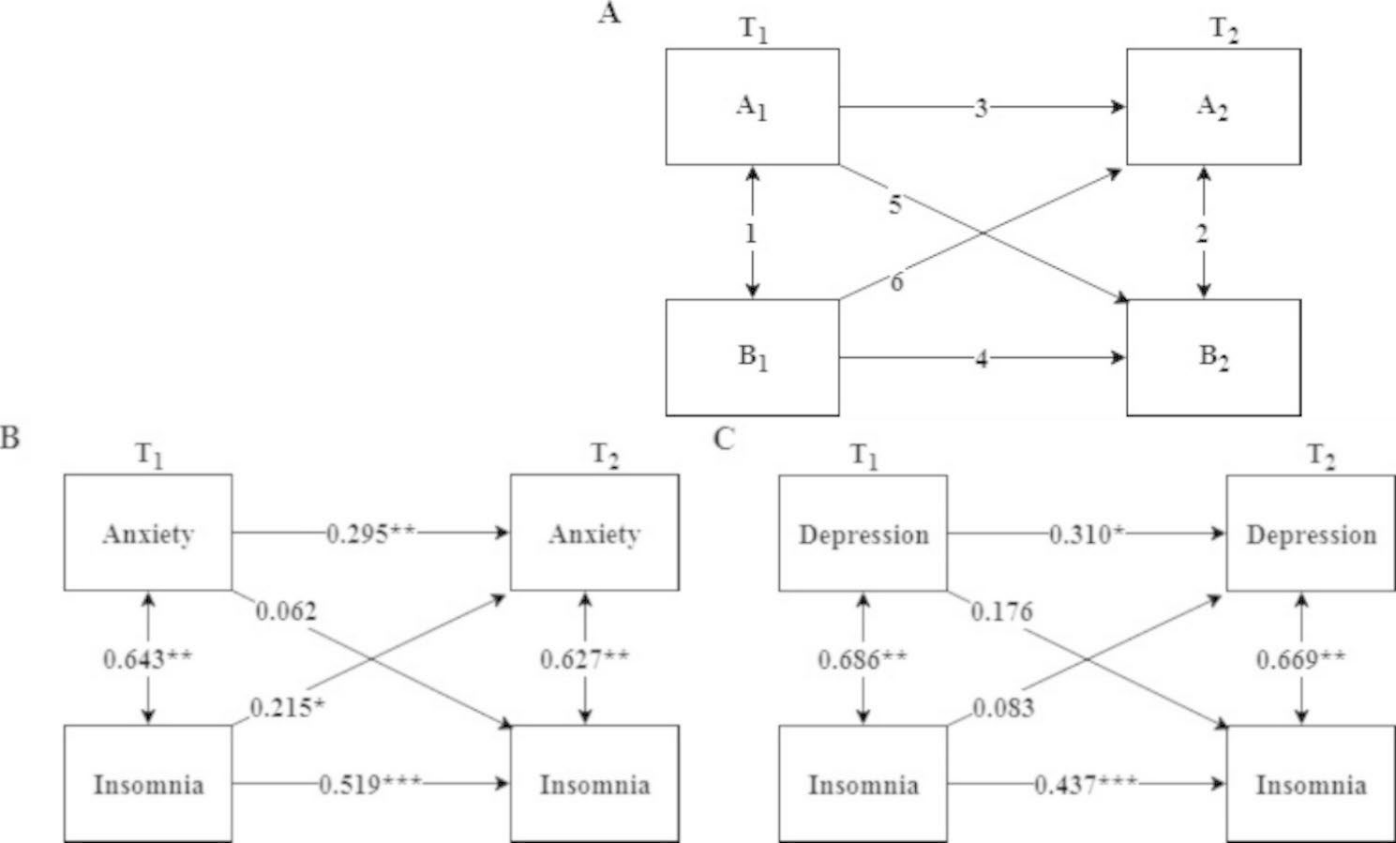
Cross-lagged model of insomnia, anxiety, and depression Note: **A**- Cross-lagged model; **B**- Cross-lagged analysis of insomnia and anxiety; **C**- Cross-lagged analysis of insomnia and depression. ^*^p < 0.05, ^**^p < 0.01, ^***^p < 0.001

Shapiro-Wilk normality tests were applied to test data distribution. The results shown that the total scores of insomnia, anxiety and depression weren’t normally distributed (*Ps* < 0.001). Therefore, comparisons of total scores of insomnia, anxiety and depression between the two time points were made using Wilcoxon tests. Pearson correlation tests were used to analyze the correlations between insomnia, anxiety, and depression in T_1_ and T_2,_ respectively. The cross-lagged analysis using regression analysis was conducted to examine prospective associations among insomnia, anxiety, and depression according to the methods described in previous research [27]. In Fig. 2A, a cross-lagged model was described in which variables A and B are measured at two time points (T_1_ and T_2_), resulting in three types of relationships: synchronous correlations (1 and 2), auto correlations (3 and 4), and cross-lagged correlations (5 and 6). The cross-lagged estimates can be interpreted as A1(B1) predicting relative changes in B2(A2). IBM SPSS 21.0 Software was used for the data analyses. *P* < 0.05, *P* < 0.01 and *P* < 0.001 were accepted as statistically significant probability values.

## Results

### Demographic characteristics

120 participants who participated in both surveys were entered into analysis. In terms of demographic characteristics, the average age of 120 subjects was 24.30 ± 4.47, and the average year of educational level was 14.03 ± 1.79.

### Longitudinal changes of insomnia, depression, and anxiety

The prevalence of insomnia, anxiety and depression were given in Table 1. Descriptive statistics and longitudinal changes of insomnia, anxiety and depression were listed in Table 2. According to the results of Wilcoxon tests, the total scores of insomnia and anxiety in T_2_ were significantly higher than that in T_1_, no significantly difference was found in depression.

**Table 1:** Prevalence of insomnia, anxiety, and depression

	Insomnia	Anxiety	Depression
T_1_	33.33%	3.33%	7.50%
T_2_	41.67%	4.17%	6.67%

**Table 2:** Descriptive statistics and longitudinal changes of insomnia, anxiety, and depression

	Insomnia	Anxiety	Depression
T_1_	4.64 ± 3.27	2.60 ± 3.37	3.18 ± 2.97
T_2_	5.25 ± 3.52	3.28 ± 2.84	3.32 ± 3.24
*Z-value*	-2.01	-2.64	-0.23
*P*	< 0.05	< 0.01	> 0.05

### Cross-sectional association of insomnia, depression, and anxiety

AIS score was significantly and positively related to the scores of GAD-7 (T_1_: *r* = 0.643, *p* < 0.01; T_2_: *r* = 0.627, *p* < 0.01) and PHQ-9 (T_1_: *r* = 0.686, *p* < 0.01; T_2_: *r* = 0.669, *p* < 0.01) in both T_1_ and T_2_, respectively.

### Cross-lagged analysis of insomnia, depression, and anxiety

The cross-lagged analysis was adopted to investigate the interactions between insomnia, anxiety, and depression after controlling the confounders (age and educational level). Firstly, hierarchical regression analysis was carried out with anxiety and insomnia scores of T_1_ as independent variables, and anxiety scores of T_2_ as dependent variables. The results showed that insomnia of T_1_ could significantly predict anxiety of T_2_, and the path coefficient was 0.215 (*p* < 0.05). Secondly, insomnia and anxiety scores of T_1_ were taken as independent variables, and insomnia scores of T_2_ as dependent variables in a new hierarchical regression model. The results indicated that the anxiety of T_1_ could not significantly predict insomnia of T_2_, and the path coefficient was 0.062 (*p* > 0.05) (Fig. 2B). Same analyses were employed to analyze the relationship between insomnia and depression. However, neither of the path coefficients (5 and 6) were significant (Fig. 2C). The results above indicated that insomnia was a predictive factor of anxiety, and there may exist a causal relationship between insomnia and anxiety only.

## Discussion

The present study selected non-clinical young Chinese males as subjects of the longitudinal study, which could avoid the potential bias of exaggerating the linkages between insomnia, anxiety and depression based on clinical participants [19]. In our longitudinal study, insomnia was significantly positively related to depression and anxiety both at baseline and follow-up. We further discovered that insomnia at baseline predicted a higher risk of anxious symptoms 8 months later. Our study may suggest a causal relationship between insomnia and anxiety, rather than insomnia and depression.

The prevalence of insomnia, anxiety, and depression at two time points (Table 1.) was consistent with previous studies. Remes and colleagues conducted a systematic review of reviews on the prevalence of anxiety disorders in adult populations, they found the prevalence of anxiety disorders in young adults range from 2.5 to 9.1% [28]. Sleep disturbances were reported by 30.5% from sample of the UK general population [29]. 6.7% of 686 male college students suffered from depression in China [13].

Consistent with previous literatures, the results revealed a significantly positive relationships among insomnia, anxiety, and depression. Prior studies indicated close relationships between insomnia and anxiety/depression. For example, Spoormaker et al. reported a high interrelatedness between sleep and depression/anxiety complaints in 402 non-clinical adults [30]. João and colleagues utilized regression model to investigate how sleep quality affected mental health in non-clinical population through 2 studies. Their first study showed that sleep quality explained 10.1% and 12.3% of the variances of depression and anxiety, respectively [18]. Compared with this study, their another research found that sleep quality explained 14.0% and 21.0% of the variances of depression and anxiety, respectively [31]. Matsumoto et al. reported consistent evidence from 90 Japanese male college students, they found poor sleepers had significantly lower scores in mental component summary (MCS) and higher scores in self-rated depression scale (SDS), indicating sleep problem was closely related to bad mental health conditions [32]. However, it is worth noting that most studies are cross-sectional and unable to infer causality.

The results of cross-lagged analysis based on our longitudinal data showed that insomnia was a predictive factor of anxiety (Fig. 2B), which indicated there existed a possible causal relationship between insomnia and anxiety. Zou et al. conducted a longitudinal observational study among 686 young Chinese male college students and found that poorer baseline sleep quality predicted a higher risk of anxious symptoms a year later [13], which was consistent with our results.

Furthermore, we would like to discuss the possible potential mechanisms underlying the predictive effect of insomnia on anxiety. Impaired executive function and dysregulated cortisol may be two neurophysiological mechanisms that may mediate the relationship between sleep disturbance and symptoms of anxiety [33]. Specifically, sleep loss may impair executive function, which will then diminish the ability to regulate or inhibit symptoms of anxiety. Besides, sleep disturbance may also lead to the dysregulated cortisol, which will contribute to the development of anxiety-related disorder. Consequently, our research suggests a possibility that anxiety can be reduced by enhancing sleep quality in non-clinical young Chinese males.

However, no predictable relationship between insomnia and depression was found in cross-lagged analysis. A potential reason of this result may be that insomnia and depression were influenced by common factor [14]. Animal studies have provided evidence for the hypothesis that common neural substrates might underlie disturbed sleep and depression [34, 35]. Moreover, the result may also relate with the scores of depression in T_1_ and T_2_. As shown in Table 2, no significantly longitudinal change of depression was found in present study.

The whole results of cross-lagged analysis were consistent with the results of a previous cohort study. Neckelmann et al. found that insomnia might be a risk factor for the development of anxiety disorders based on a 11 years interval study, but they did not find evidence suggesting that insomnia would increase the risk of developing depression [36]. What’s more, another longitudinal study reported that sleep problems in children were proved to be a predictive variable of adulthood anxiety disorders, but not depression disorders [37]. Similarly, no significant difference of the scores of depression at T_1_ and T_2_ was found in our study. Moreover, another possible explanation was proposed by researchers. Anxiety disorders were highly comorbid with depression [5]. Comorbid anxiety might confound the associations between insomnia and depression, namely a confounding effect of the comorbidity of anxiety may occur when the predictive effect of insomnia on the risk of developing depression were explored [38].

Several limitations should be noted in the present study. First of all, the sample size is relatively small. A larger sample size is needed in the further studies to investigate the relationships between insomnia and mental health problems. Secondly, the subjects of our research are all young males. Since there are gender and age differences in the prevalence of insomnia and mental health problems [39, 40], the results can’t be generalized to other population. Therefore, we should be cautious when extending our conclusions and further studies could be conducted to explore the relationships based on other population. Third, we didn’t collect demographic information such as region, occupation and so on, future study had better record these data and treat it as confounders in data analysis process. Besides, the attrition rate in the second survey was relatively large, which may limit the generalization of our study. Future researchers need to take action to decrease the subject turnover rate. Finally, although a longitudinal observational study was conducted, we still could not draw solid conclusions about causality. Thus, further randomized block design experiment and intervention studies are needed.

## Conclusion

In conclusion, we found close cross-sectional associations of insomnia with depression and anxiety. More importantly, a predictive effect of insomnia on anxiety in non-clinical young Chinese males was detected. The results suggested that insomnia might be an important cause of anxiety, while insomnia couldn’t predict depression, and vice versa.

## Electronic supplementary material

Below is the link to the electronic supplementary material.

Supplementary Material 1

## Data Availability

The datasets used and/or analysed during the current study available from the corresponding author on reasonable request.
